# Complete genome sequence of *Escherichia coli* MP1

**DOI:** 10.1128/mra.01216-23

**Published:** 2024-03-14

**Authors:** Kat Pick, Paul Stothard, Tracy L. Raivio

**Affiliations:** 1 Department of Biological Sciences, University of Alberta, Edmonton, Alberta, Canada; 2 Department of Agricultural, Food & Nutritional Science, University of Alberta, Edmonton, Alberta, Canada; University of Maryland School of Medicine, Baltimore, Maryland, USA

**Keywords:** DNA sequencing

## Abstract

Here, we report the complete genome sequence of *Escherichia coli* strain MP1, consisting of one circular chromosome and one circular plasmid. Long-read assembly was performed using a consensus approach, followed by long- and short-read polishing, and gene annotation.

## ANNOUNCEMENT

*Escherichia coli* MP1 was isolated from the feces of a healthy CD-1 mouse by selective plating on MacConkey lactose agar, then screening for beta-glucuronidase activity on LB agar containing X-Gluc, and sensitivity to phage P1_vir_ by spot titer (1). MP1 has been used as a model system to study *E. coli* colonization of the mouse gastrointestinal tract in numerous publications. The available assembly, generated by 454 pyrosequencing, consists of 84 contigs (1). We thus aimed to provide a completed genome assembly for this important model organism. MP1 was generously provided to us by the Goulian Lab (University of Pennsylvania) shipped at ambient temperature on filter paper, submerged in 5 mL LB broth upon receipt, and grown overnight at 37°C 225 rpm aerobically. Furthermore, 0.5 mL of this culture was used to create a stock in 25% glycerol, maintained at −80°C.

For DNA extraction, MP1 was streaked onto LB agar and grown overnight at 37°C. A single colony was grown overnight as above. DNA was isolated from 0.5 mL of culture using the BioSearch MasterPure Complete DNA and RNA Purification Kit following the manufacturer’s protocol, except DNA was resuspended in nuclease-free water rather than TE buffer. Illumina sequencing was performed by MiGS (Pittsburgh, PA) using the Nextera Library Preparation Kit and NextSeq 550. Default parameters were used for all software unless otherwise specified. bcl2fastq (v2.20.0.422) (Illumina) was used to demultiplex, trim adapters, and check bases and quality scores resulting in 3,024,112 read pairs (2 × 151 bp). The same DNA preparation was also sequenced by Oxford NanoPore by SeqCenter (Pittsburgh, PA) using the PCR-free Oxford Nanopore Technologies (ONT) Ligation Sequencing Kit with the NEBNext Companion Module to manufacturers’ specifications, without fragmentation or size selection. Sequencing was performed on a MinION Mk1B using R10.4.1 flow cells in a multiplexed shared flow cell run. Run design utilized the 400-bps sequencing mode with a minimum read length of 200 bp, without adaptive sampling. Guppy1 (v6.4.6) (ONT) was used for super-accurate basecalling, demultiplexing, and adapter removal (dna_r10.4.1_e8.2_400bps_modbases_5mc_cg_sup.cfg), resulting in 219,536 reads with *N*_50_ of 3,669 bp. Assembly was performed with Trycycler (v0.5.4) (2) using the procedures on the GitHub Trycycler Wiki page (3). Canu (v2.2) (4) and NextDenovo (v2.5.2) (5) were used to generate the final consensus assembly. Polishing was performed with Medaka (v1.7.2) via the Galaxy Europe server (v23.1.5.dev0) (6) using the r1041_e82_400bps_sup_g615 model and with polypolish (v0.5.0) (7) using short reads processed with fastp (v0.23.2) (8) via Galaxy (v23.1) (6).

The completed genome of MP1 consists of one circular chromosome (4,785,976 bp, 51% GC) and one circular plasmid (1) (8,555 bp, 47% GC), with an overall sequencing coverage of ~266×. Trycycler reconcile (v0.5.4) (2) was used to confirm chromosome circularity and rotate it to begin at *dnaA*. Annotation was performed by NCBI using the Prokaryotic Genome Annotation Pipeline (v6.6) (9), resulting in 4,360 protein-coding genes, 22 rRNAs, 86 tRNAs, 6 ncRNAs, 139 pseudogenes, and 1 CRISPR array.

## Data Availability

This sequencing project is deposited at NCBI BioProject PRJNA1040820. Raw reads are available at NCBI SRA; Illumina SRX22537245, Oxford NanoPore SRX22537246. The final annotated assembly is available at NCBI GenBank CP139039-CP139040.
